# Construction of the Cognitive Function Trajectory File

**DOI:** 10.1002/alz.090919

**Published:** 2025-01-09

**Authors:** Anum Zafar, Haiqun Lin, Hyosin Kim, Weiyi Xia, Maria J. Lopez, Olga F Jarrín Montaner

**Affiliations:** ^1^ Rutgers, The State University of New Jersey, New Brunswick, NJ USA; ^2^ Rutgers, The State University of New Jersey, Newark, NJ USA; ^3^ Oregon State University, Corvallis, OR USA; ^4^ Rutgers, The State University of New Jersey, Piscataway, NJ USA

## Abstract

**Background:**

Characterizing changes in cognitive function during the last years of life is vital for providing appropriate care, supporting quality of life, and planning for future demands on our medical and social resources. The aim of this project was to construct a cognitive function trajectory file spanning the last five years of life to better understand common patterns of cognitive aging.

**Method:**

The analytic cohort included 2019 Medicare decedents, aged 50 or older at death, with five years of continuous enrollment before death (n = 1,952,408). We extracted and harmonized cognitive function data elements from Medicare fee‐for‐service claims, encounter file, assessment, and Medicaid data during 2014‐2019. The data elements used for harmonization included ICD‐9 and ICD‐10 codes, the Brief Interview for Mental Status, and other cognitive function assessment items. We created a four‐category indicator for cognitive function: no impairment, memory loss, mild cognitive impairment (MCI), and dementia/severe cognitive impairment. Similar to our approach to creating the care trajectory file, the cognitive function trajectory file was expanded at day‐level and then collapsed quarterly (3‐month) basis for the last five years of life (20 quarters). We employed a thorough carry‐forward method to address gaps in information.

**Result:**

Using the information from combined assessment and claims, at death 69% of individuals aged 90 and older had dementia and 13% had MCI, and among individuals age 80‐89, 56% had dementia and 14% had MCI. Females had greater cognitive impairment at death (56% dementia, 14.4% MCI) compared to males (44.5% dementia, 15% MCI). Cognitive function at the end of life was similar across racial and ethnic groups: White (50.4% dementia, 15.1% MCI); Black (52.3% dementia, 14.7% MCI); Hispanic (51.43% dementia, 12.1% MCI); Asian American/Pacific Islander (AAPI) (52.7% dementia, 13.1% MCI); American Indian/Alaska Native (43.6% dementia, 15.2% MCI).

**Conclusion:**

The successful construction of this analytic file allows for the exploration of varying patterns of cognitive functional decline and their multi‐level predictors including individual, community, societal, and environmental factors. This approach can also be applied in research for development of new dementia care strategies and risk‐ and protective factors associated with cognitive decline.

